# Overexpression of 14-3-3θ promotes tumor metastasis and indicates poor prognosis in breast carcinoma

**DOI:** 10.18632/oncotarget.1502

**Published:** 2013-12-12

**Authors:** Nanlin Li, Hui Wang, Jing Fan, Chao Tong, Jixin Yang, Hongliang Wei, Jun Yi, Rui Ling

**Affiliations:** ^1^ Department of Vascular and Endocrine Surgery, Xijing Hospital, The Fourth Military Medical University, Xi'an, Shaanxi, China

**Keywords:** 14-3-3θ, Breast Cancer, Metastasis, Prognosis

## Abstract

An isoform of the 14-3-3 protein family, 14-3-3θ has been linked with tumor cell proliferation and apoptosis. However, the role of 14-3-3θ in the progression of breast cancer remains unknown. Here, we report that 14-3-3θ plays a critical role in breast cancer metastasis and prognosis. The expression of 14-3-3θ was markedly higher in breast cancer tissues compared to adjacent normal tissues. A hospital-based study cohort of 216 breast cancer patients was evaluated in this study. The level of 14-3-3θ expression was determined and correlated based upon tumor clinicopathological features, disease-free survival, and overall survival. We found that overexpression of 14-3-3θ was correlated with advanced TNM stage (P < 0.05), lymph node metastasis (P < 0.05), and ER negative status (P < 0.05). Breast cancer patients with high 14-3-3θ expression had a shorter overall survival and a higher rate of recurrence than those with low 14-3-3θ expression. Additionally, knockdown of 14-3-3θ expression in breast cancer cells inhibited metastasis in vitro. Similarly, an in vivo assay showed that 14-3-3θ knockdown dramatically suppressed the growth of breast cancer xenografts and inhibited tumor cell metastasis in a lung metastasis model. Thus, this study provided the first evidence that 14-3-3θ is a novel tumor suppressor and may serve as a candidate prognostic biomarker and target for new therapies in metastatic breast cancer.

## INTRODUCTION

The 14-3-3 proteins have a molecular weight of approximately 30-kDa and are a family of dimeric, well-conserved, α-helical phosphor-serine/threonine binding proteins [1]. The 14-3-3 protein family has seven mammalian isoforms (β, ε, γ, η, σ, θ, ζ), and all are able to bind to multiple protein ligands [2]. Different 14-3-3 isoforms have been implicated in the regulation of many intracellular signaling processes including mitogenesis, the DNA damage checkpoint, cell cycle control, and apoptosis via their ability to bind specific phospho-serine/threonine-containing motifs on the target protein [3]. Furthermore, the 14-3-3 proteins have been shown to regulate mitogen-activated protein kinase (MAPK) signaling by influencing the binding of Ras, Raf, and MEK, which plays a critical role in regulating tumor growth [4-6].

Compared to other 14-3-3 isoforms, few studies on 14-3-3θ have been conducted, and the studies that have evaluated the role of 14-3-3θ have all focused on cell survival and apoptosis [7, 8]. Deletion of 14-3-3θ in mice leads to embryonic lethality, and the cardiocytes of 14-3-3θ+/- mice are resistant to cardiomyocyte apoptosis [9]. Furthermore, 14-3-3θ could bind to ATM-phosphorylated E2F1 during DNA damage and promote E2F1 stability, leading to the expression of E2F1 proapoptotic target genes such as p73, Apaf1, and caspases [10]. It is reported that the 14-3-3θ isoform is bound to Bax in the cytoplasm, and Bax undergoes dissociation from 14-3-3θ during apoptosis to induce apoptotic changes in the mitochondria [11]. A recent study also showed that 14-3-3θ could inhibit tamoxifen-induced apoptosis in MCF7 breast cancer cells via interaction with p21, which is required for tamoxifen to generate a response. Additionally, it was demonstrated that 14-3-3θ expression was correlated with chemotherapy resistance in breast cancer, suggesting that 14-3-3θ is necessary for carcinogenesis and progression of human malignancies [12, 13].

To date, no studies have reported the clinicopathological significance of 14-3-3θ expression in breast cancer. In this study, we present the first evidence that 14-3-3θ expression promotes breast cancer invasion and metastasis. Additionally, we show that 14-3-3θ overexpression predicts poor prognosis in breast cancer patients after curative resection.

## RESULTS

### Expression of 14-3-3θ is significantly up-regulated in breast cancer tissues

**Figure 1:**
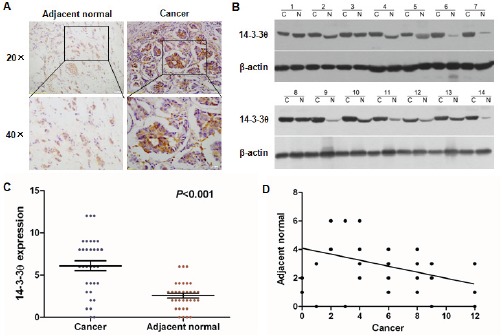
Increased 14-3-3θ expression was detected in breast cancer tissues by IHC and western blot (A) IHC staining for 14-3-3θ in human adjacent normal and breast cancer samples. Original magnification: 20× or 40×. Stronger or weaker 14-3-3θ expression was detected in cancerous or adjacent normal tissues, respectively. (B) Protein was extracted from matched breast cancer tissues and adjacent normal tissues and subjected to western blot analysis to examine 14-3-3θ expression levels. β-Actin served as a loading control. (C) Levels of 14-3-3θ expression in breast cancer or adjacent normal tissue samples. Student's t test was applied for statistical analyses. P < 0.05 was considered to be significantly different. (D) The correlation between cancerous and adjacent normal tissues and 14-3-3θ expression using linear regression.

To explore the role of 14-3-3θ in determining clinical outcomes for breast cancer patients, we first assessed 14-3-3θ protein expression in 33 pairs of human breast cancer and adjacent normal tissues by IHC analysis. IHC assays showed that expression of 14-3-3θ was primarily localized to the cytoplasm. High protein expression of 14-3-3θ was found in 21 of 33 (63.6%) primary breast cancer tissues, compared with only 3 of 33 (9.09%) adjacent normal tissues (P < 0.001) (Figure 1A and C). Up-regulation of 14-3-3θ protein expression was confirmed in an additional 14 paired breast cancer samples using western blot analysis. The levels of 14-3-3θ protein expression were significantly increased in breast cancer tissues, compared to adjacent non-tumor tissues (Figure 1B). Furthermore, a correlation study determined that 14-3-3θ protein expression in breast cancer tissue was negatively correlated with expression in adjacent normal tissues samples (P < 0.05) (Figure 1D). These results demonstrated that the expression of 14-3-3θ is increased in breast cancer, which suggested that 14-3-3θ might be involved in breast cancer tumorigenesis.

### Relationship between 14-3-3θ protein expression and clinicopathological characteristics of breast cancer

To further examine whether high protein expression of 14-3-3θ is related to the clinical progression of breast cancer, the characteristics of the 216 breast cancer patients involved in the study were examined. The expression of 14-3-3θ was detectable by IHC in all analyzed clinical specimens. The correlation between 14-3-3θ protein levels and different clinicopathological factors are shown in Table 1. No statistically significant correlations were observed between the expression of 14-3-3θ and age at diagnosis, tumor size, histological differentiation or HER2 status (P < 0.05). Statistically significant correlations between high levels of 14-3-3θ expression were found with advanced TNM stage (P = 0.013), lymph node metastasis (P < 0.0001), and ER status (P <0.0001). Spearman analysis also revealed that high 14-3-3θ expression was positively correlated with advanced TNM stage (r = 0.187, P = 0.006) and lymph node metastasis (r = 0.272, P < 0.0001) but was negatively correlated with ER status (r = -0.272, P < 0.0001) (Table 2).

**Table 1:** Statistical results of 14-3-3θ expression in 216 breast cancer specimens

Variable	No.	14-3-3 θ expression		p
		High-expression (%)	Low-expression (%)
			
Age(years)			
≤50	94	58 (61.7)	36 (38.3)	.473a
> 50	122	82 (67.2)	40 (32.8)	
Tumor size			
≤2 cm	106	68 (64.2)	38 (35.8)	.887a
> 2 cm	110	72 (65.5)	38 (34.5)	
TNM stage			
I	43	20 (46.5)	23 (53.5)	
II	65	44 (67.7)	21 (32.3)	.013b
III	64	41 (64.1)	23 (35.9)	
IV	44	35 (79.5)	9 (20.5)	
Lymph node metastasis			
Positive	130	98 (75.4)	32 (24.6)	<.0001a
Negative	86	42 (48.8)	44 (51.2)	
Histology			
Poorly differentiated	66	42 (72.8)	24 (27.2)	.599b
Moderately differentiated	63	44 (62.7)	19 (37.3)	
Well differentiated	87	54 (62.1)	33 (37.9)	
ER status			<.0001a
Positive	151	85 (24.5)	66 (75.5)	
Negative	65	55 (55.4)	10 (44.6)	
HER2 status			.529a
Positive	60	41 (68.3)	19 (31.7)	
Negative	156	99 (63.5)	57 (36.5)	

**Table 2:** The correlation between 14-3-3θ expression and clinical histopathological characteristics of 216 breast cancer specimens

Variable	14-3-3θ expression Correlation coefficient (rs)	p^a^
Age(years)	-.057	.403
Tumor size	-.014	.842
TNM stage	.187	.006
Lymph node metastasis	.272	.0001
Differentiated status	.021	.775
ER status	-.272	.0001
HER2 status	-.046	.504

a The Spearman correlation test was used for statistical analyses. P values < 0.05 were considered statistically significant.

^a^ The Spearman correlation test was used for statistical analyses. P values < 0.05 were considered statistically significant.

### High 14-3-3θ expression predicts poor prognosis in breast cancer patients

**Figure 2:**
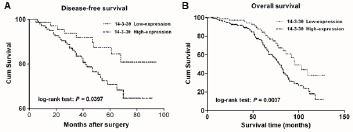
The correlation between 14-3-3θ expression and the DFS or OS of breast cancer patients Kaplan– Meier analysis of disease-free survival (A) or overall survival (B) was analyzed according to 14-3-3θ expression levels.

Kaplan–Meier analysis was used to evaluate the disease-free survival (DFS) and overall survival (OS) of patients with breast cancer according to 14-3-3θ protein expression. The results showed that patients with high expression of 14-3-3θ in breast tumor tissues had a worse DFS than those with low 14-3-3θ expression (Figure 2A, log-rank test: P = 0.0397). Breast cancer patients with high 14-3-3θ expression had a higher risk of relapse than those with low 14-3-3θ expression. Furthermore, a statistically significant association between short OS and high 14-3-3θ protein levels was found in breast cancer patients. Kaplan– Meier analysis for postoperative OS showed that breast cancer patients with high 14-3-3θ expression had a shorter OS than patients with low 14-3-3θ expression (Figure 2B, log-rank test: P = 0.0037).

### Analysis of 14-3-3θ expression in TNM stage subgroups of breast cancer patients

**Figure 3:**
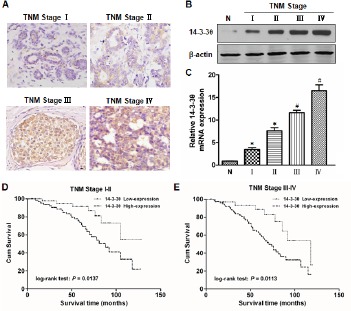
The level of 14-3-3θ expression was analyzed according to TNM stage (A) The expression of 14-3-3θ was detected in TNM stages I-IV by IHC. Original magnification: 40×. Representative images were shown. (B and C) Protein or RNA was extracted from stage I-IV breast cancer or adjacent normal tissues and subjected to western blot or real-time PCR analysis to examine the levels of 14-3-3θ expression. β-Actin served as a loading control. All data are expressed as the means ± standard deviation (SD) for three independent experiments. N (adjacent normal tissue) served as a control. * indicates P < 0.05; # indicates P < 0.01. (D and E) Kaplan–Meier analysis of DFS or OS was analyzed according to 14-3-3θ expression levels in both TNM stage I-II and III-IV.

To further investigate 14-3-3θ expression in different subgroups of breast cancer patients, 14-3-3θ expression was examined by IHC, western blot or real-time PCR assays. As shown in Figure 3A, stronger staining for 14-3-3θ was observed in breast cancer specimens through tumor stages I to IV compared to normal tissue. Western blot results showed that levels of 14-3-3θ protein were significantly increased in breast cancer tissues compared to adjacent normal tissues, and the levels increased with increasing TNM stage (Figure 3B). Similar results were found using a real-time PCR assay (Figure 3C). Additionally, the Kaplan-Meier analysis showed that OS was significantly reduced in patients with high 14-3-3θ expression versus low expression in both TNM I-II (Figure 3D, log-rank test: P = 0.0137) and TNM III-IV subgroups (Figure 3E, log-rank test: P = 0.0113).

### Analysis of 14-3-3θ expression in lymph node metastasis of breast cancer

**Figure 4:**
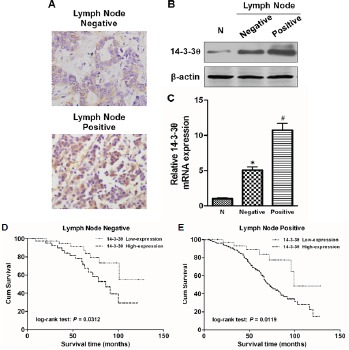
The expression of 14-3-3θ was analyzed according to lymph node metastasis status (A) The expression of 14-3-3θ was evaluated in positive and negative lymph node metastasis by IHC. The expression of 14-3-3θ was detected in both positive and negative lymph node metastasis by IHC. Original magnification: 40×. Representative images are shown. (B and C) Protein or RNA was extracted from lymph node metastasis of breast cancer patients or adjacent normal tissues and subjected to western blot or real-time PCR analysis to examine the level of 14-3-3θ expression. β-Actin served as a loading control. All data are expressed as the means ± standard deviation (SD) for three independent experiments. N (adjacent normal tissue) served as a control. * indicates P < 0.05; # indicates P < 0.01. (D and E) Kaplan–Meier analysis of DFS or OS was analyzed according to 14-3-3θ expression levels in both positive and negative lymph node metastasis breast cancer samples.

Because the above statistical analyses revealed that 14-3-3θ expression was correlated with lymph node metastasis, we further analyzed 14-3-3θ expression in different lymph node metastasis subgroups of breast cancer. IHC results showed that 14-3-3θ expression was stronger in primary breast cancer tissues if the patient also had positive lymph node metastasis (Figure 4A). Both western blot and real-time PCR data suggested that 14-3-3θ protein or RNA expression was higher in the positive lymph node group than in the negative lymph node group (Figure 4B and C). Moreover, the OS was significantly shorter in patients with high 14-3-3θ expression in both the negative lymph node metastasis subgroup (Figure 4D, log-rank test: P = 0.0312) and the positive lymph node metastasis subgroup (Figure 4E, log-rank test: P = 0.0119). These results indicated that 14-3-3θ might play a critical role in the metastatic progression of breast cancer.

### Expression of 14-3-3θ promotes breast cancer cell invasion in vitro and lung metastasis in vivo

**Figure 5:**
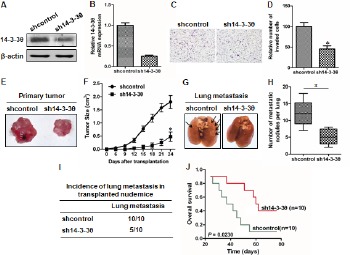
Knockdown of 14-3-3θ expression inhibited metastasis of breast cancer cells in vitro and in vivo (A and B) Protein or RNA was extracted from shcontrol or sh14-3-3θ cells and subjected to western blot or real-time PCR analysis to examine the level of 14-3-3θ expression. β-Actin served as a loading control. (C) The invasiveness of shcontrol or sh14-3-3θ cells was examined using a transwell assay. Representative images are shown. (D) Quantitative graphs are presented for the transwell assay. All data are expressed as the means ± standard deviation (SD) for three independent experiments. * indicates P < 0.05. (E) The shcontrol- or sh14-3-3θ-MDA-MB-231 cells were injected into the mammary fat pad of nude mice to evaluate tumorigenesis. Representative tumors are shown. (F) The growth curves of shcontrol or sh14-3-3θ mammary tumors are shown. * indicates P < 0.05. (G) The shcontrol- or sh14-3-3θ-MDA-MB-231 cells were injected into the lateral tail veins of nude mice to evaluate lung metastasis. Representative lungs harvested from the mice are shown. (H) The number of metastatic lung foci observed in each group is shown. * indicates P < 0.05. (I) The incidence of lung metastasis in each group of nude mice is shown. (J) Kaplan–Meier analysis of OS in each group.

To evaluate the role of 14-3-3θ in the invasion of breast cancer cells, we established two stable MDA-MB-231 cell lines (denoted shcontrol and sh14-3-3θ). Knockdown of 14-3-3θ expression was confirmed by western blot or real-time PCR analysis (Figure 5A and B). A transwell assay was used to detect differences in the metastatic ability of the two cell lines. We found that silencing endogenous 14-3-3θ expression markedly reduced MDA-MB-231 cell invasion (high initial metastatic potential) (Figure 5C and D). In addition, we injected shcontrol- or sh14-3-3θ-MDA-MB-231 cells into the mammary fat pad of nude mice and found that the growth of sh14-3-3θ-MDA-MB-231 tumors was significantly inhibited compared to the shcontrol-MDA-MB-231 cells (Figure 5E and F). We further investigated the effects of 14-3-3θ expression on breast cancer lung metastasis using MDA-MB-231 cells in vivo. An equal number of shcontrol and sh14-3-3θ breast cancer cells were injected into the lateral tail veins of nude mice to induce lung metastasis. The number of metastatic nodules in the lung was decreased in the sh14-3-3θ group compared to the shcontrol group (Figure 5H). Images of representative lung metastasis in the different groups are shown in Figure 5G. Statistical analysis further confirmed that the incidence of lung metastasis in the sh14-3-3θ group was significantly decreased compared to that of the shcontrol group (100% versus 50%) (Figure 5I). Furthermore, the sh14-3-3θ group had a longer OS time than the shcontrol group (Figure 5J). These data suggested that 14-3-3θ expression promoted the invasion and metastasis of breast cancer.

## DISCUSSION

Recurrence and metastasis remain the most common causes of mortality following curative resection of breast cancer. Thus, it is necessary to investigate the mechanisms underlying breast cancer metastasis. In this study, it was demonstrated for the first time that the expression of 14-3-3θ was frequently up-regulated in human breast cancer tissues relative to adjacent normal tissues. Overexpression of 14-3-3θ was correlated with advanced TNM stage, lymph node metastasis and ER negative status. Additionally, breast cancer patients positive for 14-3-3θ expression had a worse prognosis than patients negative for 14-3-3θ expression. These clinical data strongly suggested that 14-3-3θ contributes to the malignant progression of breast cancer and may be a useful prognostic biomarker. Several pieces of evidence in this study support a close association between 14-3-3θ expression and breast cancer metastasis. First, 14-3-3θ protein or RNA expression was markedly higher in metastatic lesions compared with their corresponding primary tumor samples. Second, down-regulation of 14-3-3θ expression significantly inhibited the invasion of breast cancer cells and their subsequent metastasis to the lungs. Although 14-3-3θprotein was first discovered from brain tissue in 1967 and it has previously been suggested that 14-3-3θ participates in the growth and apoptosis of breast cancer cells [14, 15], a direct link between breast tumorigenesis and 14-3-3θ protein expression has not yet been established. This work, therefore, represents the first study to comprehensively explore and quantify the expression levels of 14-3-3θ in a large clinical cohort of breast cancer patients. In total, there were 216 cases evaluated, and it was concluded that 14-3-3θ protein expression correlated with poor DFS and OS of breast cancer patients in our cohort. Intriguingly, high 14-3-3θ expression was associated with advanced TNM stage and lymph node metastasis in breast cancer. This finding can be explained by the functional role of 14-3-3θ. Specifically, 14-3-3θ binds to Bax and enhances Bax degradation to repress apoptosis and promote tumor cells proliferation [11, 16]. A recent study suggested that 70-75% of recurrent breast tumors have high expression of 14-3-3ζ, and primary tumors that had low levels of 14-3-3ζ expression had higher levels in the recurrence [17]. Moreover, in BT474 (both ER and HER2 positive), MCF-7-TamR (tamoxifen resistant MCF7 cells and ER positive) and MDA-MB-231 (triple negative) breast cancer cell lines, depletion of 14-3-3ζ resulted in a greatly reduced invasion capacity [17].

Although a direct link between 14-3-3θ expression and metastasis has not yet been established, our study revealed that high 14-3-3θ expression was correlated with positive lymph node metastasis, and the OS was significantly shorter in patients with high 14-3-3θ expression in both the positive and negative lymph node metastasis subgroups. Furthermore, this study showed that after knockdown of 14-3-3θ expression the invasion ability of MDA-MB-231 cells (high initial metastatic potential) was significantly suppressed in a transwell assay. Importantly, lung metastasis of MDA-MB-231 cells was also decreased. Thus, the above evidence suggested that 14-3-3θ might be a critical factor in the metastatic progression of breast cancer.

A recent pilot study using comparative 2-DE MALDI TOF/TOF MS proteomic analysis of breast tumor samples found that 14-3-3θ protein expression was associated with a positive response to neoadjuvant chemotherapy and protein levels were significantly increased in chemotherapy resistant ER-positive breast cancer [18, 19]. Notably, in this study, we found that 14-3-3θ protein expression was increased in ER-negative patients compared with ER-positive patients. In addition, low ER expression or deletion is a critical factor for chemotherapy or endocrine resistant breast cancer [20, 21]. Based on the above evidence, we speculate that a mutation or deletion of the ER might occur in breast cancer tissue with high 14-3-3θ expression, and increased expression of 14-3-3θ may suppress ER expression in breast cancer cells. Therefore, we hypothesize that 14-3-3θ expression may be a predictive biomarker of resistance to neoadjuvant chemotherapy or endocrine therapy in various stages of breast cancer progression.

In conclusion, this study demonstrates that overexpression of 14-3-3θ in breast cancer is a strong indicator of aggressive tumors and poor clinical outcome. Therefore, 14-3-3θ expression may be a candidate biomarker for breast cancer prognosis and a target for new therapies.

## MATERIALS AND METHODS

### Tissue samples and study cohort

This study was approved by the Ethics Committee of the Fourth Military Medical University. The patients who provided the 33 pairs of breast carcinoma and adjacent normal breast tissues specimens and 216 patients in the breast carcinoma sample study cohort all provided full consent for the study at the Xijing Hospital of the Fourth Military Medical University (Xi'an, China). Expression of 14-3-3θ was detected in all specimens. Tissue specimens were examined separately by 2 pathologists under double-blinded conditions without prior knowledge of the clinical status of the specimens.

### Immunohistochemistry

Immunohistochemistry (IHC) was performed using the avidin-biotin-peroxidase method on all breast carcinoma samples. All sections were deparaffinized in xylenes and dehydrated through a gradient concentration of alcohol before endogenous peroxidase activity was blocked using 0.5% H_2_O_2_ in methanol for 10 min. After non-specific binding was blocked, the slides were incubated with an anti-14-3-3θ antibody (1:200, Cell Signaling Technology, USA) in PBS at 4 °C overnight in a humidified container. Biotinylated goat anti-rabbit IgG (1:400, Sigma, USA) were incubated with the sections for 1 h at room temperature and detected using a streptavidin-peroxidase complex. Peroxidase activity was indicated by the presence of a brown color following developing with 0.1% 3,3-diaminobenzidine (Sigma) in PBS with 0.05% H_2_O_2_ for 5 min at room temperature. The appropriate positive and negative controls were included in each immunohistochemistry assay.

### Staining evaluation

An immunoreactivity score system based on the proportion and intensity of positively stained cancer cells was applied. The extensional standards that were taken were as follows: (i) number of positive stained cells ≤ 5%, scored 0; 6%-25%, scored 1; 26%-50%, scored 2; 51%-75%, scored 3; and > 75%, scored 4; and (ii) intensity of stain: colorless, scored 0; pallideflavens, scored 1; yellow, scored 2; and brown, scored 3. The extensional standards (i) and (ii) were multiplied, and the staining grade was stratified as absent (0 score), weak (1-4 score), moderate (5-8 score) or strong (9-12 score). Specimens were rescored if the difference between the scores from the 2 pathologists was greater than 3. Tumors with moderate or strong immunostaining were classified as having high expression, whereas tumors with absent or weak immunostaining were classified as having low expression.

### Measurement of disease-free survival and overall survival

Disease-free survival is defined as the time elapsed from surgery to the first occurrence of any of the following events: recurrence of breast cancer; breast cancer distant metastasis; development of second non-breast malignancy excluding basal cell carcinomas of the skin; or death from any cause without documentation of a cancer-related event. The diagnosis of recurrence and distant metastasis was based on imaging methods such as ultrasonography, computed tomography, magnetic resonance imaging, and position emission tomography and, if possible, cytologic analysis or biopsy. Overall survival is defined as the time elapsed from surgery to the time of death of the patients with breast cancer. Death of participants was ascertained by reporting from the family and verified by review of public records. The status of disease-free survival and overall survival was assigned by physicians blinded to other clinicopathologic and 14-3-3θ expression information. Follow-up data for all of the 216 patients studied were available.

### Western blot

The breast tissues were lysed in RIPA buffer (0.05 M Tris-HCl [pH 7.4], 0.15 M NaCl, 0.25% deoxycholic acid, 1% Nonidet P-40, 1 mM EDTA, 1 mM phenylmethylsulfonyl fluoride, 10 mg/ml aprotinin and 10 mg/ml leupeptin). Protein concentrations were measured using the bicinchoninic acid (BCA) protein assay (Pierce, Rockford, IL, USA). Proteins were resolved by SDS-PAGE and transferred to Hybond-ECL nitrocellulose membranes (Amersham Biosciences, Piscataway, NJ, USA). The blots were probed with various primary antibodies and species-matched secondary antibodies. The bands were detected using enhanced chemiluminescence (Pierce) or the Odyssey Imaging System (LiCor Biosciences).

### Real-time PCR

RNA was extracted from cells using Trizol reagent (Invitrogen, Life Technologies, Carlsbad, CA) and was converted into complementary DNA (cDNA) using the RevertAid First Strand cDNA Synthesis Kit (Fermentas, Foster City, CA). Real-time polymerase chain reaction (PCR) analysis was performed using the Applied Biosystems Prism 7500 Real-Time PCR Detection System (ABI, Foster City, CA) and the SYBR Premix Ex-Taq II Kit (Takara Bio, Inc., Shiga, Japan) according to the manufacturer's instructions. The relative gene expression levels were calculated using the 2-δδCt method in which Ct represents the threshold cycle, and β-actin was used as a reference gene.

### Plasmid construction and transfection

For 14-3-3θ RNA interference, the control and 14-3-3θ shRNA plasmids (sh14-3-3θ) were purchased from Santa Cruz Biotechnology (Santa Cruz, CA, USA). The expression plasmid was verified by sequencing both strands and was used to transfect MDA-MB-231 cells to establish a 14-3-3θ knockdown expression cell line.

### Transwell assay

Invasion assays were performed in 24-well transwell chambers (BD Biosciences, Bedford, MA, USA) containing polycarbonate filters with 8-mm pores coated with Matrigel (BD Biosciences). First, cells were suspended in serum-free DMEM and were added to the upper compartment of the chamber. Then, medium containing 10% FBS was added to the lower compartment of the chamber. At the indicated time points, the number of cells that had migrated through the membrane and attached to the lower surface of the membrane were counted under a light microscope for a minimum of ten random visual fields.

### In vivo tumorigenesis and metastasis assays

In total, 5×10^6^ cells were injected subcutaneously into the right mammary fat pad of 4-week-old female BALB/C athymic (nu/nu) mice (SLAC Laboratory Animal Company, Shanghai, China). Tumor length (L) and width (W) were measured every 3 days, and tumor volume was calculated using the equation: volume = (W2×L)/2. To produce the lung metastasis model, 5×10^6^ cells were injected into the lateral tail veins of 4-week-old female BALB/C athymic (nu/nu) mice. After 6 weeks, the mice were euthanized and the lung tissues were harvested for use in further experiments.

All of the experimental procedures were conducted in accordance with the Detailed Rules for the Administration of Animal Experiments for Medical Research Purposes issued by the Ministry of Health of China and received ethical approval by the Animal Experiment Administration Committee of the Fourth Military Medical University (Xi'an, P. R. China). All efforts were made to minimize the animals' suffering and reduce the number of animals used.

### Statistical analysis

All in vitro experiments were performed a total of 3 times, and each individual experiment was performed in triplicate. Data from all quantitative assays are expressed as the means ± standard deviation (S.D.) and were analyzed statistically using one-way analysis of variance, independent-samples t test or student's t test. In the clinical specimens study, associations between 14-3-3θ expression and categorical variables were analyzed by Pearson's chi-square test or Fisher's exact test, as appropriate. Correlation coefficients were analyzed by the Spearman correlation analysis. Survival curves were estimated using the Kaplan–Meier method, and differences in survival distributions were evaluated by the log-rank test. Differences with a P value of 0.05 or less were considered to be statistically significant.

The authors declare that they have no conflicts of interest. This is some
